# Association between physical activity and body fat percentage, with adjustment for BMI: a large cross-sectional analysis of UK Biobank

**DOI:** 10.1136/bmjopen-2016-011843

**Published:** 2017-03-24

**Authors:** Kathryn E Bradbury, Wenji Guo, Benjamin J Cairns, Miranda E G Armstrong, Timothy J Key

**Affiliations:** Cancer Epidemiology Unit, Nuffield Department of Population Health, University of Oxford, UK

**Keywords:** Physical activity, UK Biobank, BMI, Body fat percentage

## Abstract

**Objectives:**

The objective of this study was to examine if, in the general population, physically active adults have less body fat after taking body mass index (BMI) into account.

**Design:**

A cross-sectional analysis of participants recruited into UK Biobank in 2006–2010.

**Setting:**

UK Biobank assessment centres throughout the UK.

**Participants:**

119 230 men and 140 578 women aged 40–69 years, with complete physical activity information, and without a self-reported long-term illness, disability or infirmity.

**Exposures:**

Physical activity measured as excess metabolic equivalent (MET)-hours per week, estimated from a combination of walking, and moderate and vigorous physical activity. BMI from measured height and weight.

**Main outcome measure:**

Body fat percentage estimated from bioimpedance.

**Results:**

BMI and body fat percentage were highly correlated (r=0.85 in women; r=0.79 in men), and both were inversely associated with physical activity. Compared with <5 excess MET-hours/week at baseline, ≥100 excess MET-hours/week were associated with a 1.1 kg/m^2^ lower BMI (27.1 vs 28.2 kg/m^2^) and 2.8 percentage points lower body fat (23.4% vs 26.3%) in men, and 2.2 kg/m^2^ lower BMI (25.6 vs 27.7 kg/m^2^) and 4.0 percentage points lower body fat (33.9% vs 37.9%) in women. For a given BMI, greater physical activity was associated with lower average body fat percentage (for a BMI of 22.5–24.99 kg/m^2^: 2.0 (95% CI 1.8 to 2.2), percentage points lower body fat in men and 1.8 (95% CI 1.6 to 2.0) percentage points lower body fat in women, comparing ≥100 excess MET-hours per week with <5 excess MET-hours/week).

**Conclusions:**

In this sample of middle-aged adults, drawn from the general population, physical activity was inversely associated with BMI and body fat percentage. For people with the same BMI, those who were more active had a lower body fat percentage.

**Strengths and limitations of this study**

- This study of middle-aged adults, drawn from the general population, is very large (n=259 808) and height and weight (for the calculation of body mass index) were measured by trained staff using standardised techniques.
- Body fat percentage (estimated via bioimpedance) was available for virtually all participants.
- Physical activity was self-reported and therefore there will be some measurement error associated with this variable.
- The study is cross-sectional and therefore we cannot infer cause and effect.

## Introduction

Body mass index (BMI) is a simple index calculated from height and weight, and is usually used as a proxy for body fatness in large epidemiological studies. Correlations between BMI and more direct measures of body fatness are generally strong (r>0.70).^1–4^

Observational studies have shown that people who do comparatively more physical activity have a lower BMI than less active people.^5^ ^6^ Few large epidemiological studies have directly estimated body fatness, and it is of interest to examine whether more comprehensive measures of body fatness provide additional information above and beyond that which is captured by BMI. Previous studies, each of ∼500 young adults, have found that, for a given BMI, athletes have a lower body fat percentage than non-athletes;^7^ ^8^ however, it is unclear whether in the general population of middle-aged adults, those who do more physical activity have a lower body fat percentage than those who do minimal physical activity, after taking into account BMI.

UK Biobank is a population-based cohort of 500 000 UK men and women, aged 40–69 years at recruitment. BMI and body fat percentage were measured at recruitment for virtually all participants. For this analysis of data from UK Biobank, we aimed first to describe the associations of physical activity with BMI and body fat percentage, and second to determine whether physical activity is associated with body fat percentage, independently of BMI.

## Methods

### Subjects

UK Biobank is a prospective cohort of ∼500 000 people aged 40–69 years, recruited in 2006–2010 in the UK.^9^ People aged 40–69 years who lived within reasonable travelling distance of 22 assessment centres were identified from National Health Service patient registers and invited to participate in UK Biobank by attending an assessment centre. Permission for access to patient records for recruitment was approved by the National Information Governance Board for Health and Social Care in England and Wales, and the Community Health Index Advisory Group in Scotland. A subsample of ∼20 000 participants completed a full repeat of the assessment centre visit between August 2012 and June 2013, ∼5 years after recruitment.^10^ The UK Biobank protocol is available online (http://www.ukbiobank.ac.uk/wp-content/uploads/2011/11/UK-Biobank-Protocol.pdf). The touchscreen questionnaire and other resources are also available on the UK Biobank website (http://www.ukbiobank.ac.uk/resources/).

### Anthropometric measurements

At the UK Biobank assessment centres, a touchscreen questionnaire was used to collect information on socio-demographic characteristics and lifestyle exposures. Socks and shoes were removed and height was measured using the Seca 202 height measure (Seca, Hamburg, Germany). Weight and estimated percentage fat were measured with the Tanita BC418ma bioimpedance device (Tanita, Tokyo, Japan). Participants were not asked to fast, nor were they given any specific instructions pertaining to the bioimpedance measures prior to attending the assessment centre. Water was available at all times throughout the visit and visits occurred throughout the day (8am–8pm).

### Physical activity assessment

Questions on the touchscreen about walking, moderate physical activity and vigorous physical activity, which were similar to those used in the short form of the International Physical Activity Questionnaire,^11^ were used to estimate excess metabolic equivalent (MET)-hours/week of physical activity during work and leisure time. For each of the three activity categories (walking, moderate physical activity and vigorous physical activity), participants were asked how many days in a typical week they did each of the activities for 10 min or more (for walking: touchscreen question number WP1, UK Biobank variable n_864_0_0; for moderate physical activity: touchscreen question number WP2, UK Biobank variable n_884_0_0; and for vigorous physical activity: touchscreen question number WP3, UK Biobank variable n_904_0_0). For each category, participants who entered one or more days were then asked how many minutes they spent doing those activities on a typical day (for walking: WP1A, n_874_0_0; for moderate physical activity: WP2A, n_894_0_0; and for vigorous physical activity: WP3A, n_914_0_0). For each activity category, the number of reported days was multiplied by the number of reported minutes on a typical day to generate duration of activity in minutes per week.

Activity on a typical day of <10 min was recoded to 0 for any of the three categories of activity. For each of the three categories of activity, values of >1260 min per week (equivalent to an average of 3 hours per day) were truncated at 1260.^11^

Total MET values for each category from the International Physical Activity Questionnaire short form were: 3.3 for walking, 4.0 for moderate physical activity and 8.0 for vigorous physical activity.^11^ We report excess METs, which are calculated by subtracting one MET from the value for each activity, and represent the energy expenditure above that of an inactive person.^12^ Excess MET values were therefore 2.3 for walking, 3.0 for moderate physical activity and 7.0 for vigorous physical activity. Excess MET-hours per week were calculated by multiplying the excess MET value for each activity by the duration of activity in hours per week.^11^

### Exclusions

The UK Biobank data set used for this analysis included 502 640 participants. Participants were excluded from this analysis if they selected ‘Prefer not to answer’ or ‘Do not know’ to any of the possible six questions on physical activity (WP1, WP1A, WP2, WP2A, WP3 and WP3A) (n=66 625). Participants were also excluded from this analysis if they responded to the question: ‘Do you have a long-term illness, disability or infirmity?’ with ‘Yes’ (n=159 941), ‘Prefer not to answer’ (n=1052) or ‘Do not know’ (n=11 391), or if they had a missing value for this variable (n=919) (touchscreen question number H4, UK Biobank variable n_2188_0_0). In addition, the questions used in the pilot study on the duration of physical activity differed from those in the main study, and participants who answered the pilot version of these questions were excluded (n=2253). Based on the International Physical Activity Questionnaire recommendations for data cleaning and processing,^11^ participants were also excluded from the analysis if the sum of walking, moderate physical activity and vigorous physical activity was >112 hours per week (n=651), leaving a total of 259 808 participants in the present study.

### Statistics

STATA V.14.0 (StataCorp LP, College Station, Texas, USA) was used for all statistical analyses. All analyses were done for men and women separately. Participant characteristics were described by level of physical activity (low, <10.0; moderate, 10.0–49.9 and high, ≥50 excess MET-hours/week). Pearson's correlation coefficients between BMI and body fat percentage were calculated; values of 0.80 or above are considered very strong, values between 0.60–0.79 strong, 0.40–0.59 moderate, 0.20–0.39 weak and 0.00–0.19 very weak.^13^ Multiple linear regression was used to calculate the mean body fat percentage in single units of BMI (eg, 17.00–17.99, 18.00–18.99, 19.00–19.99 kg/m^2^, etc), adjusted for age (5-year categories: <45, 45–49.99, 50–54.99, 55–59.99, 60–64.99 and ≥65.00 years). Groups with 200 or more participants are shown in the figure. Multiple linear regression was also used to calculate mean BMI and body fat percentage in categories of excess MET-hours per week (<5, 5–9.9, 10–14.9, 15–24.9, 25–34.9, 35–49.9, 50–74.9, 75–99.9 and ≥100 excess MET-hours per week), adjusted for age (5-year categories, as above). For the final analysis, we used multiple linear regression to examine the association between physical activity (in excess MET-hours per week: <5, 5–9.9, 10–14.9, 15–24.9, 25–34.9, 35–49.9, 50–74.9, 75–99.9 and ≥100) and body fat percentage (continuous variable). BMI (in 2.5 unit categories, eg, <18.50, 18.50–19.99, 20.00–22.49, 22.50–24.99, 25.00–27.49…,42.50–44.99, ≥45.00 kg/m^2^, etc) and age (5-year categories) were included as covariates. We included a product term of excess MET-hours per week (categories, as above) and BMI (in 2.5 unit categories as above) in the model to calculate mean body fat percentage in categories of physical activity within strata of BMI. In additional sensitivity analyses, we adjusted for reported intakes of fruits and vegetables (< 3.00 servings/week, 3.00–3.99, 4.00–4.99, 5.00–5.99, ≥ 6.00 servings/week, unknown), and red and processed meat (<2.00 servings/week, 2.00–2.99, 3.00–3.99, 4.00–4.99, ≥5.00 servings/week, unknown). We also restricted the analysis to those with a university or college degree, and separately, to those who do not have a job that usually or always involves standing or walking or manual work.

We also examined mean BMI and body fat percentage in 5 year age categories. For each age decade separately (ie, participants <50 years, 50–59 years and ≥60 years) linear regression was used to calculate mean body fat percentage in single units of BMI, and to calculate mean BMI and body fat percentage in each category of physical activity (<5, 5–9.9, 10–14.9, 15–24.9, 25–34.9, 35–49.9, 50–74.9, 75–99.9 and ≥100 excess MET-hours per week).

To explore the repeatability of self-reported physical activity, including effects of measurement error and possible changes in activity over time, we used a subsample of 10 225 UK Biobank participants who were eligible for the current study and who completed a repeat assessment visit ∼5 years after recruitment (see online supplementary tables S1 and S2). For these participants, we calculated excess MET-hours per week from their answers to the touchscreen questionnaire completed at the repeat assessment centre visit, as described above. Then for each category of excess MET-hours per week defined at baseline, we calculated the mean excess MET-hours per week at their baseline visit (to assess comparability of the subsample with the full cohort) and the mean excess MET-hours per week at the repeat visit (to assess measurement error in reporting physical activity and change over time). The subsample of participants who completed a repeat assessment centre visit ∼5 years after recruitment was similar at baseline to the full cohort with regard to reported physical activity. However, at the repeat assessment, for participants in the highest category of physical activity defined at baseline (≥100 excess MET-hours per week), the mean excess MET-hours/week was much lower than at baseline (80 compared with 130 for men and women). For the lowest category of physical activity defined at baseline, the mean excess MET-hours/week was somewhat higher at the repeat assessment than at baseline (12 compared with 2.6 for men and women). Overall, this represents regression to the mean of almost 50% (calculated from the ratio of the range of mean values at the repeat assessment to the range of mean values at baseline). The Pearson's correlation coefficients between recruitment and repeat measurements of BMI and body fat percentage in the subsample of participants who completed a repeat assessment centre visit were 0.92 for both BMI and body fat percentage.

10.1136/bmjopen-2016-011843.supp1 supplementary data

All p values were two sided and p<0.05 was considered statistically significant.

## Results

**Figure 1:**
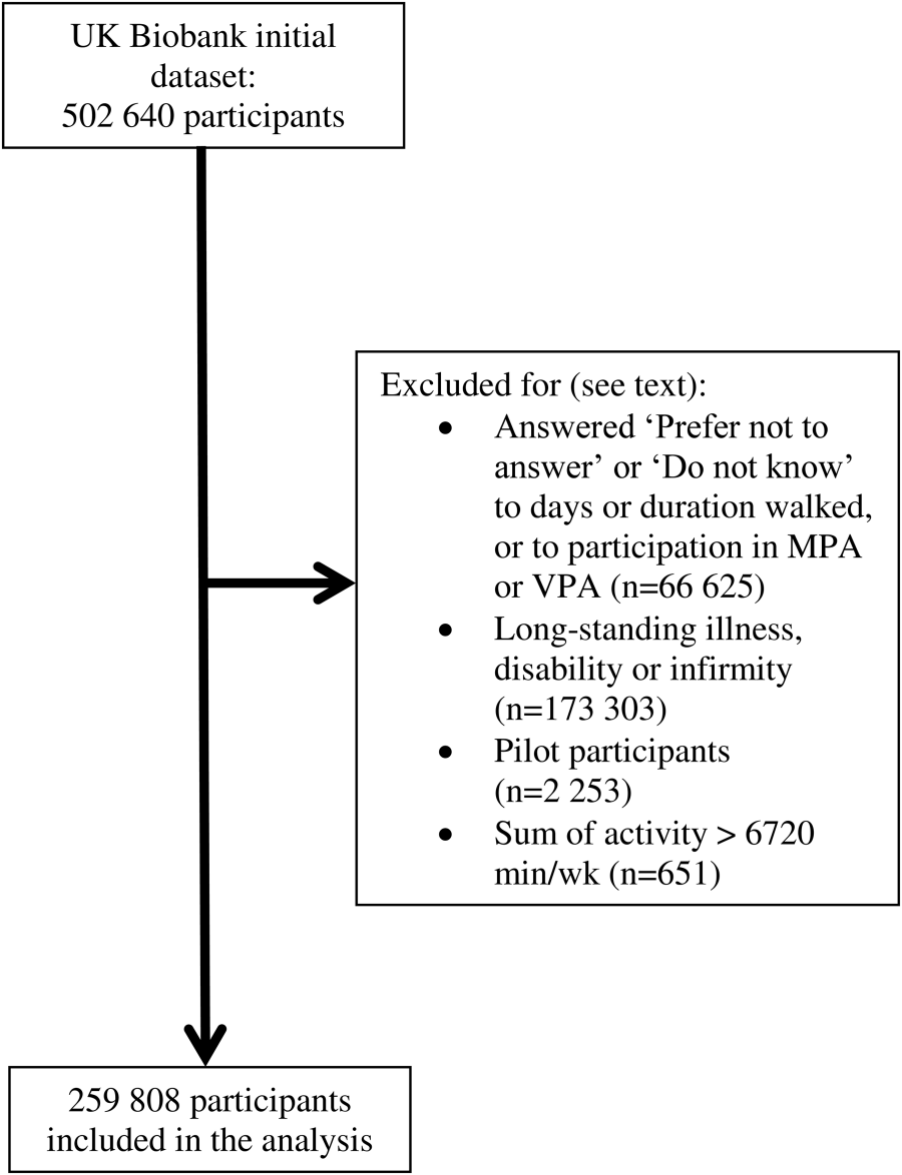
UK Biobank participant flow diagram. MPA, moderate physical activity; VPA, vigorous physical activity.

Participant flow is shown in figure 1. Participants who had a high level of physical activity were older, had a lower BMI, a lower body fat percentage and a higher intake of fruit and vegetables than participants with a low level of physical activity (table 1 and table 2). They were also less likely to have a college or university degree, and much more likely to have a standing or manual job than those with a moderate or low level of physical activity. Participants with a moderate activity level were the least likely to be current smokers.

**Table 1:** Characteristics of men participating in UK Biobank by physical activity

	Low activity	Moderate activity	High activity	
	<10 excess MET-hours/wk1	10–49.9 excess MET-hours/wk1	≥50 excess MET-hours/wk1	All men *n*=119 230
	n=26 405	n=63 022	n=29 803	
Age (years)				
Mean (SD)	55.3 (8.0)	55.7 (8.4)	56.0 (8.4)	55.7 (8.3)
White ethnicity*	24 741 (93.7)	59 842 (95.0)	28 468 (95.5)	113 051 (94.8)
Socioeconomic status†				
Upper fifth	6274 (23.8)	14 701 (23.4)	5867 (19.7)	26 842 (22.5)
Qualifications‡				
College or university degree/vocational qualification	18 878 (71.5)	45 453 (72.2)	17 731 (59.5)	82 062 (68.8)
BMI (kg/m^2^)§				
Mean (SD)	28.0 (4.2)	27.1 (3.7)	27.1 (3.6)	27.3 (3.8)
<20.00	238 (0.9)	559 (0.9)	289 (1.0)	1086 (0.9)
20.00–24.99	5849 (22.2)	17 425 (27.7)	8326 (28.0)	31 600 (26.6)
25.00–29.99	13 220 (50.2)	32 745 (52.1)	15 403 (51.8)	61 368 (51.6)
≥30.00–34.99	7015 (26.7)	12 135 (19.3)	5699 (19.2)	24 849 (20.9)
Body fat (%)¶				
Mean (SD)	25.8 (5.5)	24.2 (5.4)	23.6 (5.5)	24.4 (5.5)
<15.00	761 (2.9)	3062 (4.9)	1945 (6.6)	5768 (4.9)
15.00–19.99	2810 (10.8)	9714 (15.6)	5148 (17.5)	17 672 (15.0)
20.00–24.99	7714 (29.6)	21 023 (33.7)	10 080 (34.3)	38 817 (32.9)
25.00–29.99	9194 (35.2)	19 816 (31.8)	8686 (29.5)	37 696 (32.0)
≥30.00	5629 (21.6)	8703 (14.0)	3558 (12.1)	17 890 (15.2)
Height (m)**				
<1.70	3902 (14.8)	9251 (14.7)	5467 (18.4)	18 620 (15.7)
1.70–1.74	6250 (23.8)	15 301 (24.4)	7917 (26.6)	29 468 (24.8)
1.75–1.79	7738 (29.4)	18 182 (28.9)	8246 (27.8)	34 166 (28.7)
1.80–1.84	5308 (20.2)	12 992 (20.7)	5408 (18.2)	23 708 (19.9)
≥1.85	3115 (11.8)	7120 (11.3)	2682 (9.0)	12 917 (10.9)
Mean (SD) excess MET-hours per week††	5.1 (2.9)	25.8 (11.0)	92.9 (43.3)	38.0 (40.1)
Standing or walking job‡‡	2524 (12.7)	9766 (23.1)	12 961 (65.4)	25 251 (30.8)
Manual job§§	561 (2.8)	3214 (7.6)	8145 (41.1)	11 920 (14.6)
Smoking status¶¶				
Never	14 269 (54.0)	34 209 (54.3)	15 101 (50.7)	63 579 (53.3)
Previous	8866 (33.6)	22 438 (35.6)	10 936 (36.7)	42 240 (35.4)
Current	3215 (12.2)	6252 (9.9)	3680 (12.4)	13 147 (11.0)
Alcohol consumption***				
Three or more times a week	14 391 (54.5)	36 542 (58.0)	15 477 (52.0)	66 410 (55.8)
Fruit and vegetable consumption†††				
<3.00 servings per day	8754 (33.2)	14 052 (22.3)	5966 (20.0)	28 772 (24.1)
3.00–3.99 servings per day	6031 (22.8)	13 370 (21.2)	5204 (17.5)	24 605 (20.6)
4.00–4.99 servings per day	4619 (17.5)	12 622 (20.0)	5485 (18.4)	22 726 (19.1)
5.00–5.99 servings per day	2926 (11.1)	8930 (14.2)	4389 (14.7)	16 245 (13.6)
≥6.00 servings per day	3798 (14.4)	13 559 (21.5)	8405 (28.2)	25 762 (21.6)
Total red and processed meat consumption‡‡‡				
<2.00 times per week	2120 (8.0)	5838 (9.3)	2956 (9.9)	10 914 (9.2)
2.00–2.99 times a week	6291 (23.8)	16 042 (25.5)	7170 (24.1)	29 503 (24.7)
3.00–3.99 times a week	3927 (14.9)	9323 (14.8)	4186 (14.1)	17 436 (14.6)
4.00–4.99 times a week	4866 (18.4)	10 990 (17.4)	5069 (17.0)	20 925 (17.6)
≥5.00 times a week	9042 (34.2)	20 523 (32.6)	10 229 (34.3)	39 794 (33.4)

Values are number (%) unless otherwise stated.

Number of participants with missing data (the total number participants who have missing data, or who reported ‘do not know’ or ‘prefer not to answer’) for each characteristic is as follows: 0 for age, 377 for ethnicity, 159 for socioeconomic status, 705 for qualifications, 327 for BMI, 1367 for body fat %, 351 for height, 0 for excess MET-hours/week, 57 for standing or walking job, 38 for manual job, 264 for smoking status, 35 for alcohol consumption, 1120 for fruit and vegetable consumption and 658 for total red and processed meat consumption.

*Participants who reported their ethnicity as ‘White’, ‘British’, ‘Irish’ or ‘Any other white background’.

†We generated quintiles of socioeconmic status based on the Townsend deprivation index for the whole cohort (UK Biobank variable n_189_0_0).

‡Vocational qualifications defined as other professional qualification (eg: nursing or teaching/ national vocational qualification or higher national diploma or higher national certificate) (touchscreen question number D12, UK Biobank variable n_6138_0_0).

§We preferentially used BMI derived from height and weight measured during the impedance measurement (UK Biobank variable n_23 104 _0_0_), but if missing, used the body size measures (UK Biobank variable n_21 001 _0_0); both of these are direct measures of height and weight made on the same day at the assessment centre.

¶Body fat % (UK Biobank variable n_23 099 _0_0).

**Standing height (UK Biobank variable n_50_0_0).

††Excess MET-hours/wk estimated from the combination of reported walking, moderate and vigorous physical activity (for details see methods text).

‡‡Participants who reported their work ‘usually’ or ‘always’ involved walking or standing for most of the time (touchscreen question number D9B, UK Biobank variable n_806_0_0).

§§Participants who reported their work ‘usually’ or ‘always’ involved heavy manual or physical work for most of the time (touchscreen question number D9C, UK Biobank variable n_816_0_0).

¶¶Smoking status (UK Biobank variable n_20 116 _0_0).

***Participants who reported consuming alcohol three to four times per week or daily or almost daily (touchscreen question number A1, UK Biobank variable n_1558_0_0).

†††Total fruit and vegetable consumption is the sum of fresh fruit intake (touchscreen question number DT3, UK Biobank variable n_1309_0_0), cooked vegetable intake (touchscreen question numbers DT1, UK Biobank variable n_1289_0_0) and raw vegetable intake (touchscreen question number DT1, and UK Biobank variable n_1299_0_0). To sum the frequencies, ‘Less than one’ was coded as 0.5, and we coded one piece of fresh fruit as a serving and two tablespoons of vegetables as a serving.

‡‡‡Total red and processed meat consumption is the sum of processed meat (touchscreen question number DT8 and UK Biobank variable n_1349_0_0), beef (touchscreen question number DT7 and UK Biobank variable n_1369_0_0), lamb/mutton (touchscreen question number DT7A and UK Biobank variable n_1379_0_0) and pork (touchscreen question number DT7B and UK Biobank variable n_1389) intake. To sum the frequencies, we used the following coding: ‘Never’=0, ‘Less than once a week’ =0.5, ‘Once a week’=1, ‘2–4 times a week’=3, ‘5–6 times a week’=5.5, ‘Once or more daily’=7.

BMI, body mass index; MET, metabolic equivalent.

**Table 2:** Characteristics of women participating in UK Biobank by physical activity

	Low activity	Moderate activity	High activity	
	<10 excess MET-hours/wk1	10–49.9 excess MET-hours/wk1	≥50 excess MET-hours/wk1	All women *n*=140 578
	n=31 931	n=78 171	n=30 476	
Age (years)				
Mean (SD)	54.6 (7.8)	55.2 (8.1)	56.2 (8.1)	55.3 (8.1)
White ethnicity*	30 164 (94.5)	74 471 (95.3)	29 088 (95.5)	133 723 (95.1)
Socioeconomic status†				
Upper fifth	6956 (21.8)	17 177 (22.0)	6407 (21.0)	30 540 (21.8)
Qualifications‡				
College or university degree/vocational qualification	19 320 (60.5)	49 219 (62.9)	17 826 (58.5)	86 365 (61.5)
BMI (kg/m^2^)§				
Mean (SD)	27.2 (5.1)	26.0 (4.4)	25.7 (4.2)	26.2 (4.6)
<20.00	954 (3.0)	2838 (3.6)	1270 (4.2)	5062 (3.6)
20.00–24.99	10 930 (34.3)	33 256 (42.7)	13 749 (45.2)	57 935 (41.3)
25.00–29.99	12 037 (37.8)	29 041 (37.2)	11 062 (36.4)	52 140 (37.2)
≥30.00–34.99	7930 (24.9)	12 839 (16.5)	4334 (14.3)	25 103 (17.9)
Body fat (%)¶				
Mean (SD)	37.1 (6.6)	35.2 (6.5)	34.4 (6.6)	35.5 (6.7)
<25.00	1197 (3.8)	4842 (6.3)	2524 (8.4)	8563 (6.2)
25.00–29.99	3256 (10.3)	11 220 (14.5)	4920 (16.3)	19 396 (14.0)
30.00–34.99	7011 (22.2)	20 467 (26.5)	8224 (27.3)	35.702 (25.7)
35.00–39.99	9129 (29.0)	22 181 (28.7)	8347 (27.7)	39 657 (28.6)
≥40.00	10 940 (34.7)	18 506 (24.0)	6110 (20.3)	35 556 (25.6)
Height (m)**				
<1.55	2758 (8.7)	6315 (8.1)	2810 (9.2)	11 883 (8.5)
1.55–1.59	6268 (19.7)	15 551 (19.9)	6504 (21.4)	28 323 (20.2)
1.60–1.64	10 067 (31.6)	24 437 (31.3)	9631 (31.7)	44 135 (31.5)
1.65–1.69	7993 (25.1)	20 091 (25.8)	7530 (24.8)	35 614 (25.4)
≥1.70	4779 (15.0)	11 621 (14.9)	3952 (13.0)	20 352 (14.5)
Mean (SD) excess MET-hours per week††	5.3 (2.8)	25.5 (10.9)	83.0 (33.3)	33.4 (32.5)
Standing or walking job‡‡	3901 (17.4)	12 349 (25.7)	9593 (56.3)	25 843 (29.53)
Manual job§§	521 (2.3)	2576 (5.4)	3883 (22.8)	6980 (8.0)
Smoking status¶¶				
Never	19 513 (61.1)	47 974 (61.4)	18 370 (60.3)	85 857 (61.1)
Previous	9468 (29.7)	24 366 (31.2)	9635 (31.6)	43 469 (30.9)
Current	2883 (9.0)	5661 (7.2)	2417 (7.9)	10 961 (7.8)
Alcohol consumption***				
Three or more times a week	12 670 (39.7)	33 747 (43.2)	12 137 (39.8)	58 554 (41.7)
Fruit and vegetable consumption†††				
<3.00 servings per day	6601 (20.7)	9849 (12.6)	3070 (10.1)	19 520 (13.9)
3.00–3.99 servings per day	6507 (20.4)	13 485 (17.3)	4232 (13.9)	24 224 (17.2)
4.00–4.99 servings per day	6672 (20.9)	16 489 (21.1)	5701 (18.7)	28 862 (20.5)
5.00–5.99 servings per day	5092 (16.0)	14 460 (18.5)	5573 (18.3)	25 125 (17.9)
≥6.00 servings per day	6874 (21.5)	23 557 (30.1)	11 740 (38.5)	42 171 (30.0)
Total red and processed meat consumption‡‡‡				
<2.00 times per week	5462 (17.1)	15 254 (19.5)	6729 (22.1)	27 445 (19.5)
2.00–2.99 times a week	10 903 (34.2)	27 329 (35.0)	10 190 (33.4)	48 422 (34.4)
3.00–3.99 times a week	5395 (16.9)	12 642 (16.2)	4823 (15.8)	22 860 (16.3)
4.00–4.99 times a week	4058 (12.7)	9143 (11.7)	3423 (11.2)	16 624 (11.8)
≥5.00 times a week	5973 (18.7)	13 456 (17.2)	5144 (16.9)	24 573 (17.5)

Values are number (%) unless otherwise stated.

Number of participants with missing data (the total number participants who have missing data, or who reported ‘do not know’ or ‘prefer not to answer’) for each characteristic is as follows: 0 for age, 263 for ethnicity, 150 for socioeconomic status, 756 for qualifications, 338 for BMI, 1704 for body fat %, 271 for height, 0 for excess MET-hours/wk, 64 for standing or walking job, 52 for manual job, 291 for smoking status, 44 for alcohol consumption, 676 for fruit and vegetable consumption, 655 for total red and processed meat consumption.

*Participants who reported their ethnicity as ‘White’, ‘British’, ‘Irish’ or ‘Any other white background’.

†We generated quintiles of socioeconmic status based on the Townsend deprivation index for the whole cohort (UK Biobank variable n_189_0_0).

‡Vocational qualifications defined as other professional qualification (eg. nursing or teaching)/ National Vocational Qualification or Higher National Diploma or Higher National Certificate) (touchscreen question number D12, UK Biobank variable n_6138_0_0).

§We preferentially used BMI derived from height and weight measured during the impedance measurement (UK Biobank variable n_23 104 _0_0_), but if missing used the body size measures (UK Biobank variable n_21 001 _0_0); both of these are direct measures of height and weight made on the same day at the assessment centre.

¶Body fat % (UK Biobank variable n_23 099 _0_0).

**Standing height (UK Biobank variable n_50_0_0).

††Excess MET-hours/wk estimated from the combination of reported walking, moderate and vigorous physical activity (for details see methods text).

‡‡Participants who reported their work ‘usually’ or ‘always’ involved walking or standing for most of the time (touchscreen question number D9B, UK Biobank variable n_806_0_0).

§§Participants who reported their work ‘usually’ or ‘always’ involved heavy manual or physical work for most of the time (touchscreen question number D9C, UK Biobank variable n_816_0_0).

¶¶Smoking status (UK Biobank variable n_20 116 _0_0).

***Participants who reported consuming alcohol three to four times per week or daily or almost daily (touchscreen question number A1, UK Biobank variable n_1558_0_0).

†††Total fruit and vegetable consumption is the sum of fresh fruit intake (touchscreen question number DT3, UK Biobank variable n_1309_0_0), cooked vegetable intake (touchscreen question numbers DT1, UK Biobank variable n_1289_0_0) and raw vegetable intake (touchscreen question number DT1, and UK Biobank variable n_1299_0_0). To sum the frequencies, ‘Less than one’ was coded as 0.5, and we coded 1 piece of fresh fruit as a serving and 2 tablespoons of vegetables as a serving.

‡‡‡Total red and processed meat consumption is the sum of processed meat (touchscreen question number DT8 and UK Biobank variable n_1349_0_0), beef (touchscreen question number DT7 and UK Biobank variable n_1369_0_0), lamb/mutton (touchscreen question number DT7A and UK Biobank variable n_1379_0_0), and pork (touchscreen question number DT7B and UK Biobank variable n_1389) intake. To sum the frequencies, we used the following coding: ‘Never’=0, ‘Less than once a week’=0.5, ‘Once a week’=1, ‘2–4 times a week’=3, ‘5–6 times a week’=5.5, ‘Once or more daily’=7.

BMI, body mass index; MET, metabolic equivalent.

**Figure 2:**
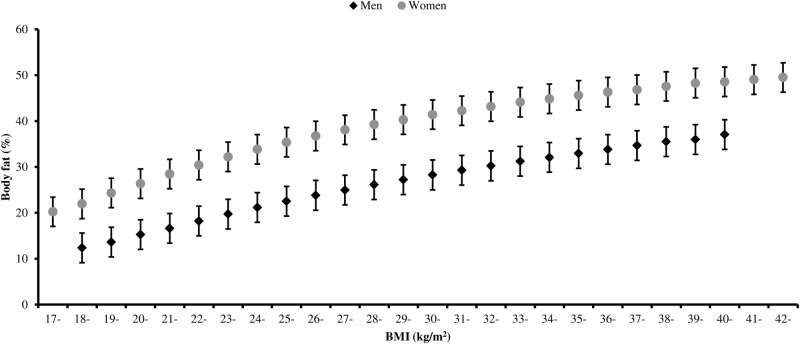
Body fat percentage by BMI in UK Biobank. Values are mean body fat percentage by single-unit BMI categories. Adjusted for age (5-year categories). Error bars represent one SD either side of the mean. Estimates shown for cells with 200 or more participants. BMI, body mass index.

Body fat percentage was positively related to BMI (figure 2). The correlation between BMI and body fat percentage was very strong in women (r=0.85), and strong in men (r=0.79). At the same BMI, women had a much higher body fat percentage than men; for example, women with a BMI of 30.00–30.99 kg/m^2^ had on average 41% body fat, whereas men with the same BMI had on average 28% body fat.

**Figure 3:**
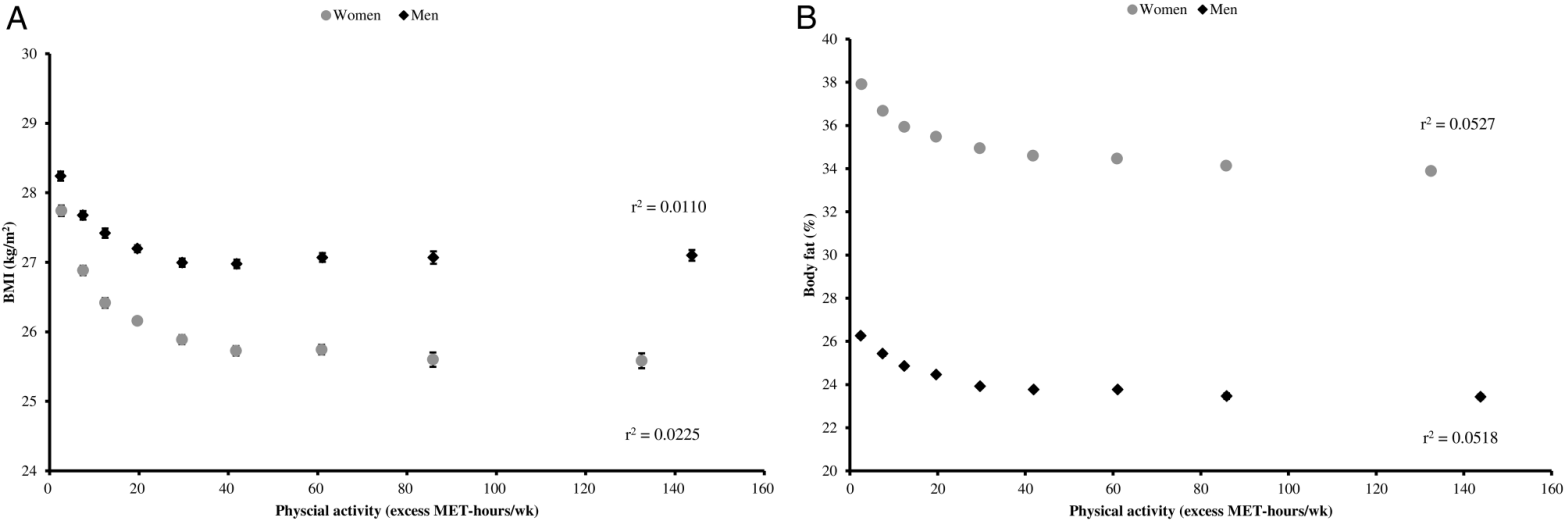
Mean BMI and body fat percentage by physical activity in UK Biobank. Panel A: mean BMI by physical activity (excess MET-hours/wk); panel B: mean body fat percentage by physical activity (excess MET- hours/wk). Values are mean BMI and body fat percentage in the following categories of physcial activity: <5, 5–9.9, 10–14.9, 15–24.9, 25–34.9, 35–49.9, 50–74.9, 75–99.9 and ≥100 excess MET-hrs per week, and are plotted at the value of the mean excess MET-hours/wk in each category. Adjusted for age (5-year categories). Errors bars are 95% CI. Estimates shown for cells with 200 or more participants. BMI, body mass index; MET, metabolic equivalent.

Body fat percentage and BMI were inversely related to physical activity (figure 3). Men who did <5 excess MET-hours of physical activity per week had, on average, a BMI of 28.2 kg/m^2^ (95% CI 28.2 to 28.3 kg/m^2^) and 26.3% (95% CI 26.2 to 26.4%) body fat. Men who did ≥100 excess MET-hours per week of physical activity per week had, on average, a BMI of 27.1 kg/m^2^ (95% CI: 27.0 to 27.2 kg/m^2^) and 23.4% (95% CI 23.3 to 23.5%) body fat. Women who did <5 excess MET-hours per week of physical activity per week had, on average, a BMI of 27.7 kg/m^2^ (95% CI 27.7 to 27.8 kg/m^2^) and 37.9% (95% CI 37.8 to 38.0%) body fat. Women who did ≥100 excess MET-hours/week of physical activity per week had, on average, a BMI of 25.6 kg/m^2^ (95% CI 25.5 to 25.7 kg/m^2^) and a 33.9% (95% CI 33.7 to 34.0%) body fat. For men and women, as shown by the r^2^ values, age and physical activity explained more of the variation in body fat percentage than they did the variation in BMI in this study population; however, age and physical activity only explained a small proportion of the variation in BMI and body fat percentage in this study population, with all r^2^ values <0.06 (figure 3).

**Figure 4:**
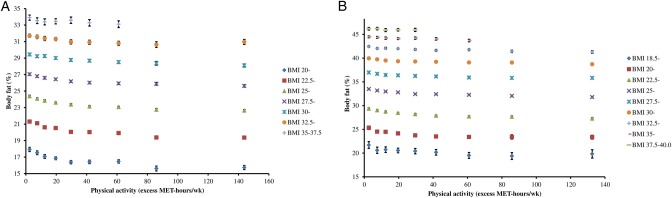
Mean body fat percentage by physical activity, stratified by BMI category in UK Biobank. Panel A: men; panel B: women. Values are mean body fat percentage in the following categories of physical activity: <5, 5–9.9, 10–14.9, 15–24.9, 25–34.9, 35–49.9, 50–74.9, 75–99.9, ≥100 excess MET-hrs per week, and are plotted at the value of the mean excess MET-hours/wk in each category. Estimates shown for cells with 200 or more participants. Adjusted for age (5-year categories). BMI, body mass index; MET, metabolic equivalent.

Overall, in men, those doing ≥100 or more excess MET-hours/week compared with <5 excess MET-hours/week had a 1.7 (95% CI 1.6 to 1.7) percentage points lower body fat percentage, on average, after adjustment for BMI and age; in women it was on average 1.5 (95% CI 1.4 to 1.6) percentage points lower. For men and women, within each stratum of BMI, a higher physical activity level was associated with a lower body fat percentage, and the difference in body fat percentage between physical activity categories appeared to be slightly larger at lower BMIs (p for interaction using likelihood ratio test <0.001, for both sexes) (figure 4, see online supplementary tables S3 and S4). For a BMI of 22.5–24.99 kg/m^2^, ≥100 excess MET-hours per week versus <5 excess MET-hours per week was associated with 2.0 (95% CI 1.8 to 2.2) percentage points lower body fat in men and 1.9 (95% CI 1.6 to 2.0) percentage points lower body fat in women. For men and women, within each stratum of BMI, the mean BMI was very similar across the categories of physical activity.

When we further adjusted for reported intakes of fruit and vegetables, and red and processed meats, or restricted the analysis to those who did not have an active job or those who had a university or college degree, the results were not materially altered. Comparing those doing ≥100 excess MET-hours per week with those doing < 5 excess MET-hours per week, body fat percentage was, on average 1.6 (95% CI 1.5 to 1.7) percentage points lower for men and 1.4 (95% CI 1.3 to 1.5) percentage points lower for women after adjustment for diet quality; 1.7 (95% CI 1.6 to 1.9) percentage points lower for men and 1.6 (95% CI 1.4 to 1.8) percentage points lower for women when restricting analyses to those who had a university or college degree; and 1.6 (95% CI 1.5 to 1.7) percentage points lower for men and 1.4 (95% CI 1.3 to 1.5) percentage points lower for women when we restricted analyses to those who did not have a standing or manual job.

In men, the mean BMI was similar across 5-year age categories; however, the mean body fat percentage was higher in older age groups. In women, the mean BMI by 5-year age categories was slightly higher in older age groups, and the mean body fat percentage was also higher in older age groups (see online supplementary table S5). The association between BMI and body fat percentage was similar in each age decade (see online supplementary figures S1, S2 and S3). The differences in BMI between the extreme categories of physical activity were slightly larger and the differences in body fat percentage were slightly smaller with older age (see online supplementary figures S4, S5 and S6).

## Discussion

In this large sample of middle-aged British men and women, more physical activity was associated with a lower BMI and a lower body fat percentage, although even men and women who did the most physical activity were, on average, overweight. More physical activity was also associated with a lower body fat percentage within each category of BMI, with an average 1–2 percentage points lower body fat in the most active, compared with the least active individuals. Most of the difference in body fat percentage with physical activity was between the very low and moderately high levels of physical activity (<5 and 35–49.9 excess MET-hours per week, respectively); there was relatively little difference in body fat percentage between moderately high and very high levels of physical activity (35–49.9 and ≥100 excess MET-hours per week, respectively).

The current study is large, and height and weight were measured by trained staff using standardised techniques. We examined whether important lifestyle factors (diet quality, education and job type) which varied by physical activity level might modify the associations between physical activity, BMI and body fat percentage. In each of these sensitivity analyses, the results were essentially unchanged, although because this is an observational study we cannot rule out confounding by other factors. A limitation of the study is that physical activity was self-reported. Analysis of the subsample who had a repeat measurement of physical activity ∼5 years after baseline indicates ∼50% regression to the mean, which represents the error in reporting physical activity and true changes in physical activity over time. The likely consequence of regression to the mean in physical activity levels over time is bias of associations towards the null, so that the true association between physical activity and body composition measures is likely to be stronger than that observed in this study. Participants were not given any specific instructions prior to body fat measurement. Hydration status, exercise and food consumption can have small effects on body fat values measured by bioimpedance; had these factors been standardised between participants, we may have seen slightly stronger associations between body fat percentage and physical activity. The study is cross-sectional, and therefore we can only show associations between reported physical activity and contemporaneous body composition. We cannot infer cause and effect: lower levels of physical activity may lead to greater adiposity, but it is also possible that increased adiposity leads to less physical activity.

Previous small studies (n<200), in young athletic populations have found inverse relationships between measures of physical fitness and BMI and body fat percentage.^14^ ^15^ Small studies (n∼500) in young adults have also shown that, for a given BMI, athletes have a lower body fat percentage than non-athletes.^7^ ^8^ These findings are, however, of limited relevance to older adults in the general population, who experience the highest burden of obesity-related disease. An analysis of 466 605 participants in the China Kadoorie Biobank, aged 30–79 years, found relatively weak associations between physical activity and either BMI or body fat percentage: a difference of ∼100 total MET-hours per week was associated with 0.15 kg/m^2^ lower BMI, and 0.48 percentage points lower body fat.^16^ Participants in the China Kadoorie Biobank differed from those in UK Biobank in ethnicity and lifestyle, and also had a lower average BMI (23.4 (SD 3.2) kg/m^2^ in men; 23.8 (SD 3.4) kg/m^2^ in women). Their physical activity levels were comparable with the middle to upper range of physical activity of UK Biobank participants, and in this range we also saw only a small difference in body fat percentage.

Variation in BMI in the general population is largely due to differences in body fatness, but by definition it incorporates adipose and lean body mass, and it is therefore difficult to disentangle the roles of adipose and lean mass in associations of BMI with health outcomes. For example, a higher BMI is an established risk factor for postmenopausal breast cancer, and probably increases risk through higher circulating sex hormones produced by the enzyme aromatase in the adipose tissue from precursor androgens.^17^ Several cohort studies have also shown that more physical activity is associated with a reduced risk of postmenopausal breast cancer, even after adjustment for BMI, and this finding is often taken as evidence that physical activity is independent of adiposity as a risk factor for postmenopausal breast cancer.^18^ Our results suggest, however, that adjustment for BMI may not have fully controlled for adiposity in these analyses.

In conclusion, in this sample of middle-aged British adults who were free from self-reported long-standing illness, men and women who reported doing the most physical activity had a lower BMI and a lower body fat percentage than those who reported doing the least physical activity. We also report new evidence that, for a given BMI, men and women who reported doing more physical activity had a lower body fat percentage; the greatest difference was observed between low and moderate levels of physical activity. BMI incorporates adipose and lean mass, but is most strongly related to adiposity, and consequently is associated with morbidity and mortality from a wide range of diseases. However, to disentangle the possible effects of physical activity and adiposity on disease risk, future research should focus on more specific measures of adiposity.
