# Supplementary material for: Association between physical activity and body fat percentage, with adjustment for BMI: a large cross-sectional analysis of UK Biobank
Source: BMJ Open. 2017 Mar 24;7(3):e011843. doi: 10.1136/bmjopen-2016-011843 (PMC5372047; doi:10.1136/bmjopen-2016-011843)
Supplement: supplementary data [file bmjopen-2016-011843supp.pdf]

**Table S1** Repeated measures of physical activity in men who completed the repeat assessment visit in UK Biobank 5 years after recruitment

| | Physical activity (excess MET-hours per week)[a] | | | | | | | | |
|---|---|---|---|---|---|---|---|---|---|
| | <5 | 5-9.9 | 10-14.9 | 15-24.9 | 25-34.9 | 35-49.9 | 50-74.9 | 75-99.9 | ≥ 100 |
| | *All men (n = 119,230)* | | | | | | | | |
| Number of men | *12,613* | *13,792* | *12,381* | *20,983* | *15,023* | *14,635* | *13,415* | *7,043* | *9,345* |
| Baseline[b] | 2.5 | 7.5 | 12.4 | 19.7 | 29.7 | 41.9 | 61.1 | 85.9 | 143.8 |
| | *Men who completed the repeat assessment visit (n = 5,158)* | | | | | | | | |
| Number of men | *602* | *614* | *534* | *943* | *691* | *631* | *541* | *279* | *323* |
| Baseline[b] | 2.6 | 7.6 | 12.4 | 19.7 | 29.8 | 42.1 | 60.5 | 85.6 | 133.3 |
| Repeat[b] | 12.2 | 16.3 | 20.8 | 26.1 | 32.2 | 40.3 | 47.9 | 60.5 | 82.8 |

[a]Categories are defined from baseline data

[b]Values are mean excess MET-hours per week

**Table S2** Repeated measures of physical activity in women who completed the repeat assessment visit in UK Biobank 5 years after recruitment

| | Physical activity (excess MET-hours per week)[a] | | | | | | | | |
|---|---|---|---|---|---|---|---|---|---|
| | <5 | 5-9.9 | 10-14.9 | 15-24.9 | 25-34.9 | 35-49.9 | 50-74.9 | 75-99.9 | ≥ 100 |
| | *All women (n = 140,578)* | | | | | | | | |
| Number of women | *14,299* | *17,632* | *15,970* | *26,156* | *18,555* | *17,490* | *16,231* | *7,407* | 6,838 |
| Baseline[b] | 2.6 | 7.5 | 12.4 | 19.6 | 29.6 | 41.7 | 60.9 | 85.8 | 132.5 |
| | *Women who completed the repeat assessment visit (n = 5,067)* | | | | | | | | |
| Number of women | *515* | *659* | *606* | *1,004* | *681* | *640* | *555* | *229* | *178* |
| Baseline[b] | 2.6 | 7.6 | 12.3 | 19.6 | 29.7 | 41.7 | 61.0 | 85.0 | 133.0 |
| Repeat[b] | 11.9 | 18.5 | 21.3 | 26.1 | 33.2 | 40.6 | 52.8 | 59.2 | 80.7 |

[a]Categories are defined from baseline data

[b]Values are mean excess MET-hours per week

**Table S3** Mean (95% CI) body fat percentage by categories of BMI and physical activity in men in UK Biobank.

| Categories of BMI | Categories of physical activity (MET-hours/wk) | | | | | | | | |
|---|---|---|---|---|---|---|---|---|---|
| | <5 | 5-9.9 | 10-14.9 | 15-24.9 | 25-34.9 | 35-49.9 | 50-74.9 | 75-99.9 | ≥ 100 |
| 20- | 16.6 (16.3-16.8) *n = 649* | 16.1 (15.9-16.4) *n = 780* | 15.7 (15.4-15.9) *n = 743* | 15.4 (15.3-15.6) *n = 1356* | 14.9 (14.7-15.1) *n = 1014* | 15.0 (14.7-15.2) *n = 986* | 15.0 (14.8-15.2) *n = 940* | 14.1 (13.8-14.4) *n = 478* | 14.3 (14.0-14.6) *n = 658* |
| *Mean BMI* | *21.6* | *21.5* | *21.5* | *21.5* | *21.5* | *21.5* | *21.6* | *21.5* | *21.5* |
| 22.5- | 19.9 (19.8-20.1) *n = 1943* | 19.7 (19.6-19.8) *n = 2477* | 19.2 (19.0-19.3) *n = 2411* | 19.1 (19.0-19.2) *n = 4289* | 18.6 (18.5-18.7) *n = 3379* | 18.6 (18.4-18.7) *n = 3247* | 18.4 (18.3-18.6) *n = 2851* | 17.9 (17.7-18.1) *n = 1490* | 17.9 (17.8-18.1) *n = 1909* |
| *Mean BMI* | *23.9* | *23.9* | *23.9* | *23.9* | *23.9* | *23.9* | *23.8* | *23.9* | *23.8* |
| 25- | 23.0 (22.9-23.1) *n = 3217* | 22.7 (22.6-22.8) *n = 3861* | 22.4 (22.3-22.5) *n = 3606* | 22.2 (22.1-22.3) *n = 6180* | 21.9 (21.8-22.0) *n = 4428* | 21.7 (21.6-21.8) *n = 4419* | 21.6 (21.5-21.7) *n = 3960* | 21.3 (21.1-21.4) *n = 2095* | 21.2 (21.1-21.3) *n = 2694* |
| *Mean BMI* | *26.2* | *26.2* | *26.2* | *26.2* | *26.2* | *26.2* | *26.2* | *26.2* | *26.2* |
| 27.5- | 25.7 (25.6-25.8) *n = 2950* | 25.4 (25.3-25.5) *n = 3192* | 25.2 (25.1-25.3) *n = 2793* | 25.0 (24.9-25.1) *n = 4818* | 24.7 (24.6-24.9) *n = 3302* | 24.6 (24.4-24.7) *n = 3199* | 24.5 (24.3-24.6) *n = 2948* | 24.4 (24.2-24.6) *n = 1561* | 24.2 (24.0-24.3) *n = 2145* |
| *Mean BMI* | *28.6* | *28.6* | *28.6* | *28.6* | *28.6* | *28.6* | *28.6* | *28.6* | *28.6* |
| 30- | 28.1 (27.9-28.2) *n = 1867* | 27.8 (27.7-28.0) *n = 1780* | 27.8 (27.7-28.0) *n = 1498* | 27.6 (27.4-27.7) *n = 2409* | 27.3 (27.2-27.5) *n = 1644* | 27.2 (27.1-27.4) *n = 1585* | 27.1 (26.9-27.2) *n = 1560* | 26.9 (26.7-27.1) *n = 809* | 26.7 (26.5-26.9) *n = 1107* |
| *Mean BMI* | *31.1* | *31.1* | *31.1* | *31.0* | *31.0* | *31.0* | *31.0* | *31.0* | *31.0* |
| 32.5- | 30.4 (30.2-30.6) *n = 984* | 30.2 (30.0-30.4) *n = 873* | 30.0 (29.7-30.2) *n = 663* | 29.9 (29.7-30.1) *n = 1009* | 29.5 (29.3-29.8) *n = 654* | 29.5 (29.2-29.7) *n = 634* | 29.3 (29.1-29.6) *n = 614* | 29.2 (28.8-29.6) *n = 335* | 29.5 (29.2-29.8) *n = 440* |
| *Mean BMI* | *33.6* | *33.6* | *33.5* | *33.5* | *33.4* | *33.5* | *33.5* | *33.5* | *33.5* |

| | | | | | | | | | |
|---|---|---|---|---|---|---|---|---|---|
| 35-<br>*Mean BMI* | 32.5<br>(32.2-32.9)<br>*n = 428*<br>*36.1* | 32.1<br>(31.8-32.5)<br>*n = 358*<br>*36.0* | 32.0<br>(31.6-32.4)<br>*n = 302*<br>*36.0* | 32.0<br>(31.7-32.3)<br>*n = 416*<br>*36.0* | 32.2<br>(31.8-32.5)<br>*n = 280*<br>*36.0* | 31.8<br>(31.4-32.2)<br>*n = 261*<br>*36.0* | 31.7<br>(31.3-32.1)<br>*n = 247*<br>*36.0* | - | - |
| 37.5-<br>*Mean BMI* | 34.3<br>(33.9-34.8)<br>*n = 217*<br>*38.5* | - | - | - | - | - | - | - | - |

Adjusted for age (5 year categories)
Values not shown for cells with less than 200 participants

**Table S4** Mean (95% CI) body fat percentage by categories of BMI and physical activity in women in UK Biobank

| Categories of BMI | Categories of physical activity (MET-hours/wk) | | | | | | | | |
|---|---|---|---|---|---|---|---|---|---|
| | <5 | 5-9.9 | 10-14.9 | 15-24.9 | 25-34.9 | 35-49.9 | 50-74.9 | 75-99.9 | ≥ 100 |
| 18.5- | 24.4 | 23.5 | 23.5 | 23.1 | 22.7 | 22.5 | 22.3 | 22.3 | 22.3 |
| | (24.0-24.8) | (23.2-23.9) | (23.2-23.8) | (22.9-23.4) | (22.4-23.0) | (22.2-22.7) | (22.0-22.6) | (21.9-22.8) | (21.9-22.7) |
| | *n = 313* | *n = 460* | *n = 405* | *n = 749* | *n = 569* | *n = 556* | *n = 526* | *n = 219* | *n = 267* |
| *Mean BMI* | *19.4* | *19.3* | *19.4* | *19.4* | *19.4* | *19.4* | *19.4* | *19.3* | *19.4* |
| 20- | 28.4 | 28.0 | 27.8 | 27.5 | 27.2 | 26.8 | 26.7 | 26.6 | 26.2 |
| | (28.2-28.6) | (27.9-28.2) | (27.6-27.9) | (27.4-27.6) | (27.1-27.3) | (26.7-27.0) | (26.5-26.8) | (26.4-26.8) | (26.0-26.4) |
| | *n = 1657* | *n = 2333* | *n = 2378* | *n = 4095* | *n = 3115* | *n = 3034* | *n = 2746* | *n = 1327* | *n = 1156* |
| *Mean BMI* | *21.4* | *21.4* | *21.4* | *21.4* | *21.4* | *21.4* | *21.4* | *21.4* | *21.4* |
| 22.5- | 32.6 | 32.2 | 32.0 | 31.8 | 31.4 | 31.4 | 31.2 | 31.0 | 30.7 |
| | (32.4-32.7) | (32.1-32.3) | (31.9-32.1) | (31.7-31.9) | (31.3-31.5) | (31.3-31.5) | (31.1-31.3) | (30.8-31.2) | (30.6-30.9) |
| | *n = 2872* | *n = 4068* | *n = 4014* | *n = 6819* | *n = 4981* | *n = 4820* | *n = 4496* | *n = 2026* | *n = 1998* |
| *Mean BMI* | *23.8* | *23.7* | *23.7* | *23.7* | *23.7* | *23.7* | *23.7* | *23.7* | *23.7* |
| 25- | 36.0 | 35.7 | 35.4 | 35.4 | 35.2 | 35.1 | 34.9 | 34.8 | 34.8 |
| | (35.9-36.2) | (35.6-35.8) | (35.3-35.6) | (35.3-35.5) | (35.1-35.3) | (35.0-35.2) | (34.8-35.0) | (34.6-35.0) | (34.6-35.0) |
| | *n = 2953* | *n = 4016* | *n = 3594* | *n = 6034* | *n = 4288* | *n = 4069* | *n = 3705* | *n = 1692* | *n = 1516* |
| *Mean BMI* | *26.2* | *26.1* | *26.1* | *26.1* | *26.1* | *26.1* | *26.1* | *26.1* | *26.1* |
| 27.5- | 39.0 | 38.8 | 38.5 | 38.3 | 38.3 | 38.2 | 38.1 | 38.0 | 37.7 |
| | (38.9-39.2) | (38.6-38.9) | (38.4-38.7) | (38.2-38.5) | (38.2-38.4) | (38.1-38.4) | (37.9-38.2) | (37.8-38.2) | (37.5-37.9) |
| | *n = 2353* | *n = 2715* | *n = 2370* | *n = 3704* | *n = 2616* | *n = 2366* | *n = 2262* | *n = 1008* | *n = 879* |
| *Mean BMI* | *28.6* | *28.6* | *28.6* | *28.6* | *28.6* | *28.6* | *28.6* | *28.6* | *28.6* |
| 30- | 41.5 | 41.1 | 41.1 | 41.0 | 40.8 | 40.6 | 40.8 | 40.4 | 40.3 |
| | (41.3-41.7) | (40.9-41.3) | (40.9-41.3) | (40.9-41.2) | (40.6-41.0) | (40.4-40.8) | (40.6-41.0) | (40.1-40.7) | (40.0-40.6) |
| | *n = 1625* | *n = 1716* | *n = 1424* | *n = 2185* | *n = 1366* | *n = 1237* | *n = 1181* | *n = 564* | *n = 468* |
| *Mean BMI* | *31.1* | *31.1* | *31.1* | *31.1* | *31.1* | *31.0* | *31.1* | *31.0* | *31.1* |
| 32.5- | 43.6 | 43.4 | 43.2 | 43.1 | 43.2 | 43.0 | 42.7 | 42.7 | 42.5 |

| | | | | | | | | | |
|---|---|---|---|---|---|---|---|---|---|
| | (43.4-43.8) | (43.2-43.6) | (43.0-43.5) | (42.9-43.3) | (43.0-43.5) | (42.8-43.3) | (42.4-43.0) | (42.3-43.1) | (42.0-42.9) |
| | *n = 1008* | *n = 989* | *n = 774* | *n = 1149* | *n = 699* | *n = 651* | *n = 611* | *n = 260* | *n = 233* |
| *Mean BMI* | *33.6* | *33.6* | *33.6* | *33.6* | *33.6* | *33.5* | *33.5* | *33.5* | *33.5* |
| | 45.3 | 45.2 | 44.9 | 45.0 | 44.9 | 44.9 | 44.8 | | |
| 35- | (45.0-45.5) | (45.0-45.5) | (44.6-45.3) | (44.7-45.3) | (44.6-45.3) | (44.5-45.3) | (44.4-45.2) | | |
| | *n = 577* | *n = 577* | *n = 444* | *n = 584* | *n = 359* | *n = 313* | *n = 282* | - | - |
| *Mean BMI* | *36.1* | *36.1* | *36.0* | *36.1* | *36.1* | *36.1* | *36.0* | | |
| | 47.0 | 46.6 | 46.8 | 46.7 | 46.6 | | | | |
| 37.5- | (46.6-47.3) | (46.2-47.0) | (46.3-47.2) | (46.3-47.1) | (46.1-47.0) | | | | |
| | *n = 368* | *n = 303* | *n = 207* | *n = 316* | *n = 205* | - | - | - | - |
| *Mean BMI* | *38.6* | *38.5* | *38.6* | *38.5* | *38.5* | | | | |
| | 48.3 | | | | | | | | |
| 40- | (47.8-48.7) | | | | | | | | |
| | *n = 220* | - | - | - | - | - | - | - | - |
| *Mean BMI* | *41.1* | | | | | | | | |

Adjusted for age (5 year categories)

Values not shown for cells with less than 200 participants

**Table S5** BMI and body fat percentage by sex and 5-year age category in UK Biobank

| | *n* | BMI | Body fat percentage |
|---|---|---|---|
| Men | | | |
| < 45 years | 15176 | 27.2 (4.0) | 22.8 (5.5) |
| 45-49 years | 17569 | 27.4 (3.9) | 23.5 (5.5) |
| 50-54 years | 18375 | 27.5 (3.9) | 24.1 (5.5) |
| 55-59 years | 20580 | 27.3 (3.8) | 24.6 (5.5) |
| 60-64 years | 26532 | 27.3 (3.7) | 25.1 (5.4) |
| ≥ 65 years | 20671 | 27.1 (3.6) | 25.6 (5.3) |
| Women | | | |
| < 45 years | 17392 | 25.7 (4.8) | 33.7 (7.0) |
| 45-49 years | 21848 | 26.0 (4.8) | 34.3 (7.0) |
| 50-54 years | 23728 | 26.3 (4.7) | 35.3 (6.7) |
| 55-59 years | 25329 | 26.3 (4.6) | 35.9 (6.5) |
| 60-64 years | 31600 | 26.5 (4.4) | 36.4 (6.3) |
| ≥ 65 years | 20343 | 26.5 (4.2) | 36.7 (6.0) |

Values are mean (SD)

Numbers of participants shown are for BMI within each age category, slightly fewer participants have information on body fat percentage

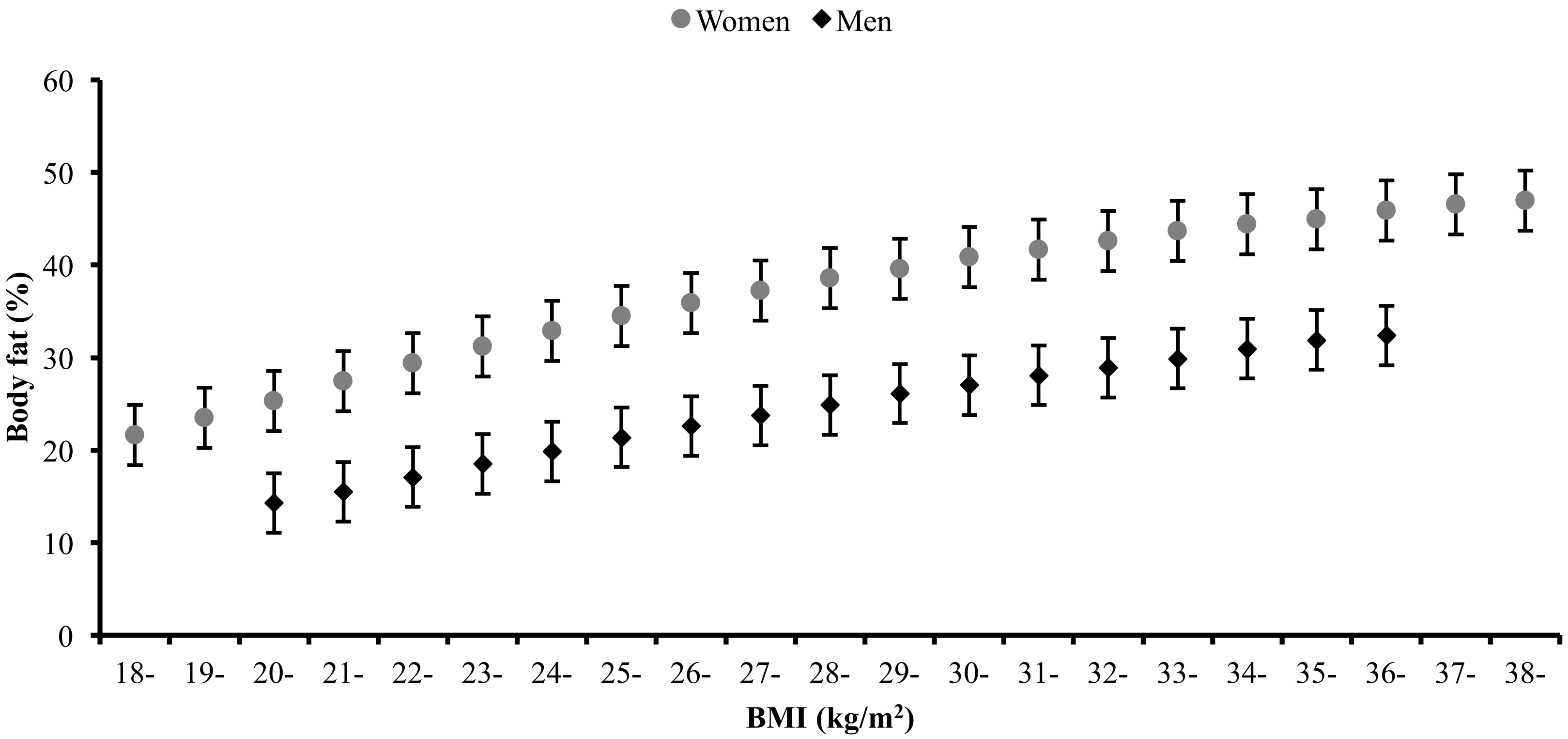


**Figure S1** Body fat percentage by BMI for participants aged younger than 50 years in UK Biobank
Values are mean body fat percentage by single-unit BMI categories
Error bars represent 1 SD either side of the mean
Estimates shown for cells with 200 or more participants

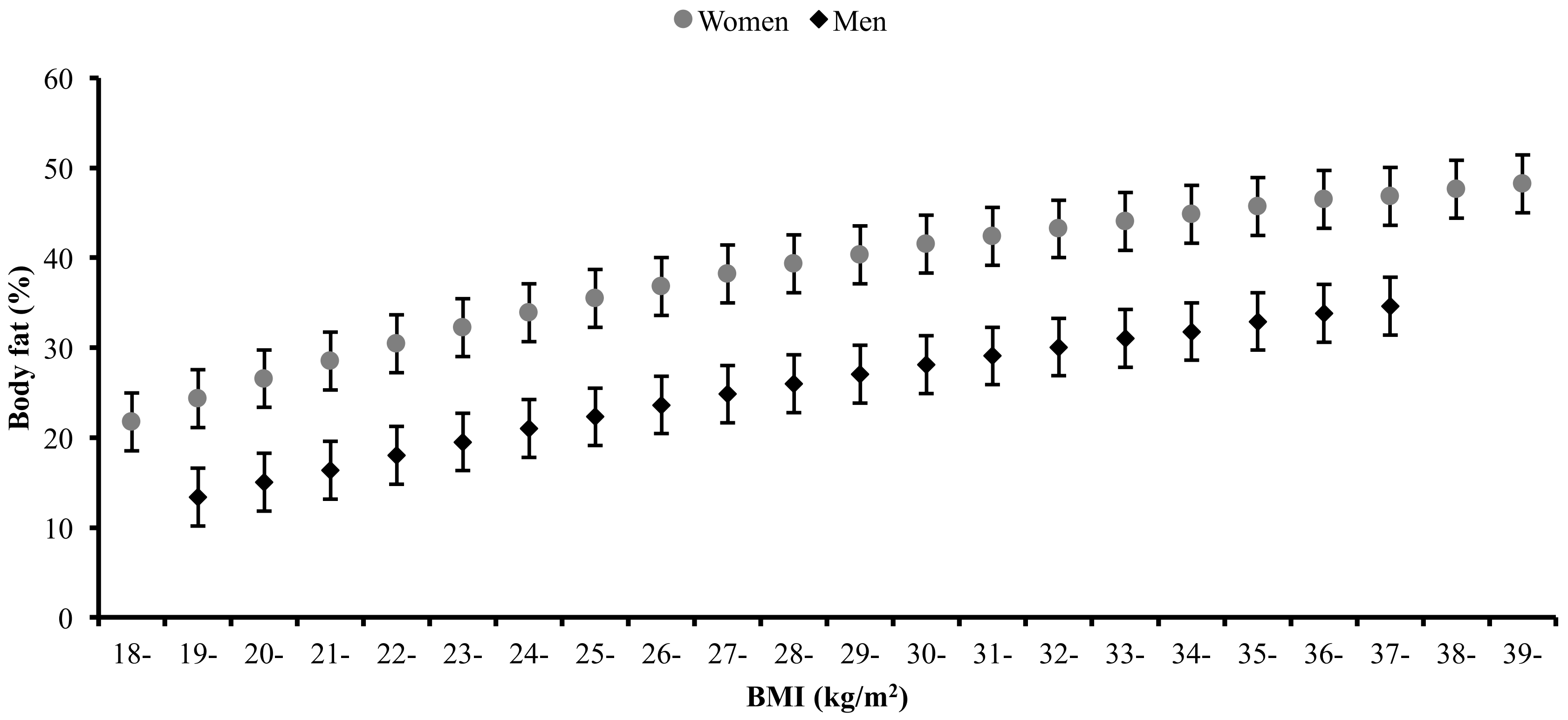


**Figure S2** Body fat percentage by BMI for participants aged 50-59 years in UK Biobank
Values are mean body fat percentage by single-unit BMI categories
Error bars represent 1 SD either side of the mean
Estimates shown for cells with 200 or more participants

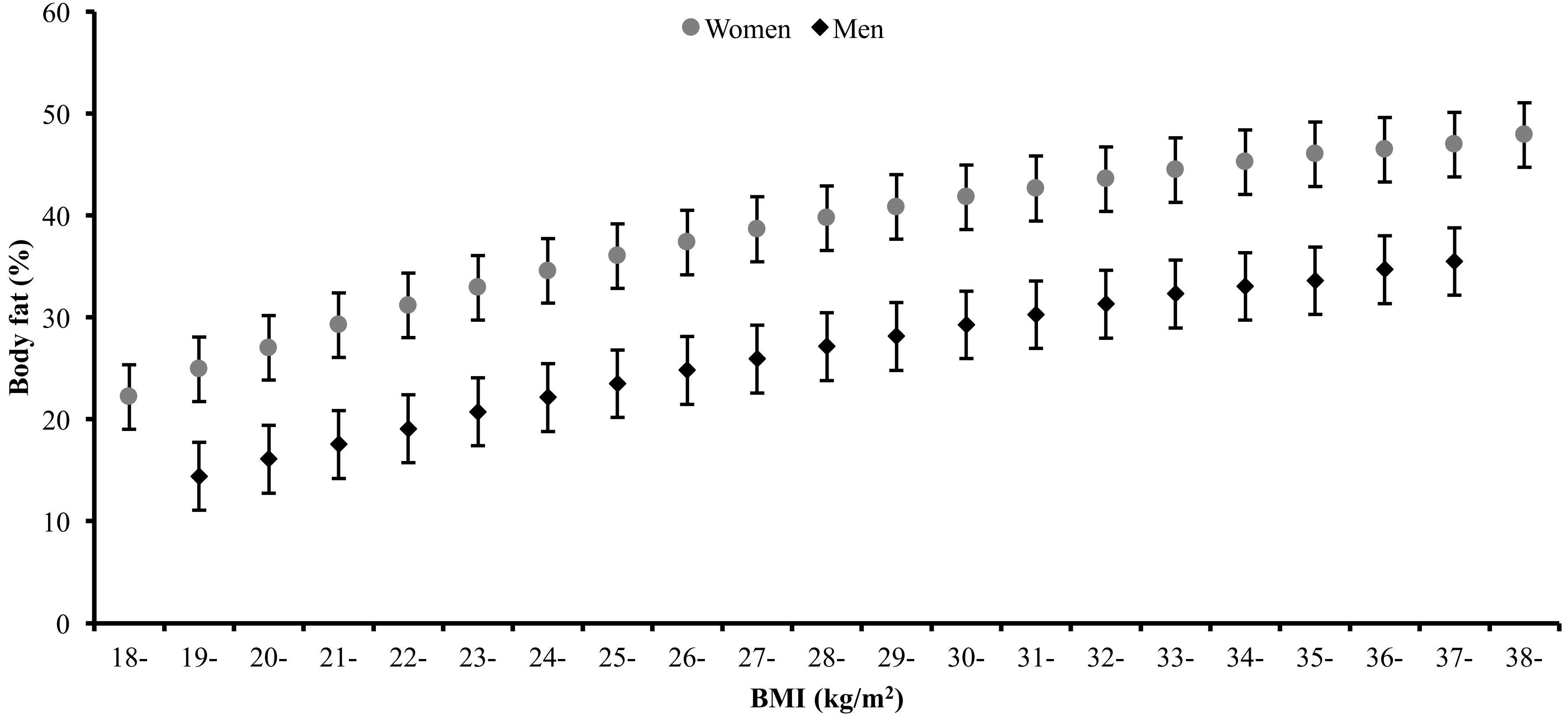


**Figure S3** Body fat percentage by BMI for participants aged 60 years or older in UK Biobank
Values are mean body fat percentage by single-unit BMI categories
Error bars represent 1 SD either side of the mean
Estimates shown for cells with 200 or more participants

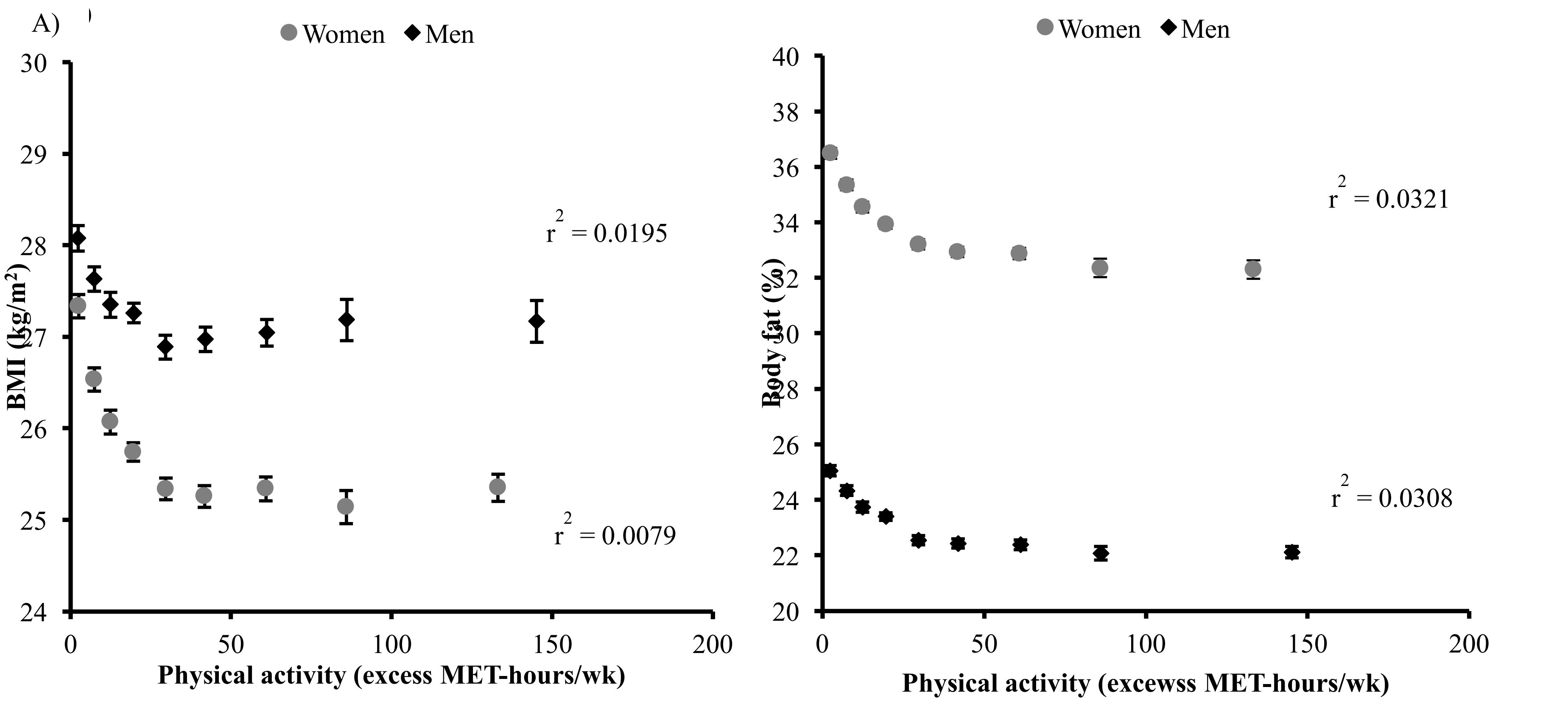


**Figure S4** Mean BMI and body fat percentage by physical activity for participants aged younger than 50 years in UK Biobank

Panel A: Mean BMI by physical activity (excess MET-hours/wk); Panel B: Mean body fat percentage by physical activity (excess MET-hrs/wk)

Values are mean BMI and body fat percentage in the following categories of physcial activity: <5, 5-9.9, 10-14.9, 15-24.9, 25-34.9, 35-49.9, 50-74.9, 75-99.9, ≥ 100 excess MET-hrs per week, and are plotted at the value of the mean excess MET-hours/wk in each category

Errors bars are 95% CI

Estimates shown for cells with 200 or more participants

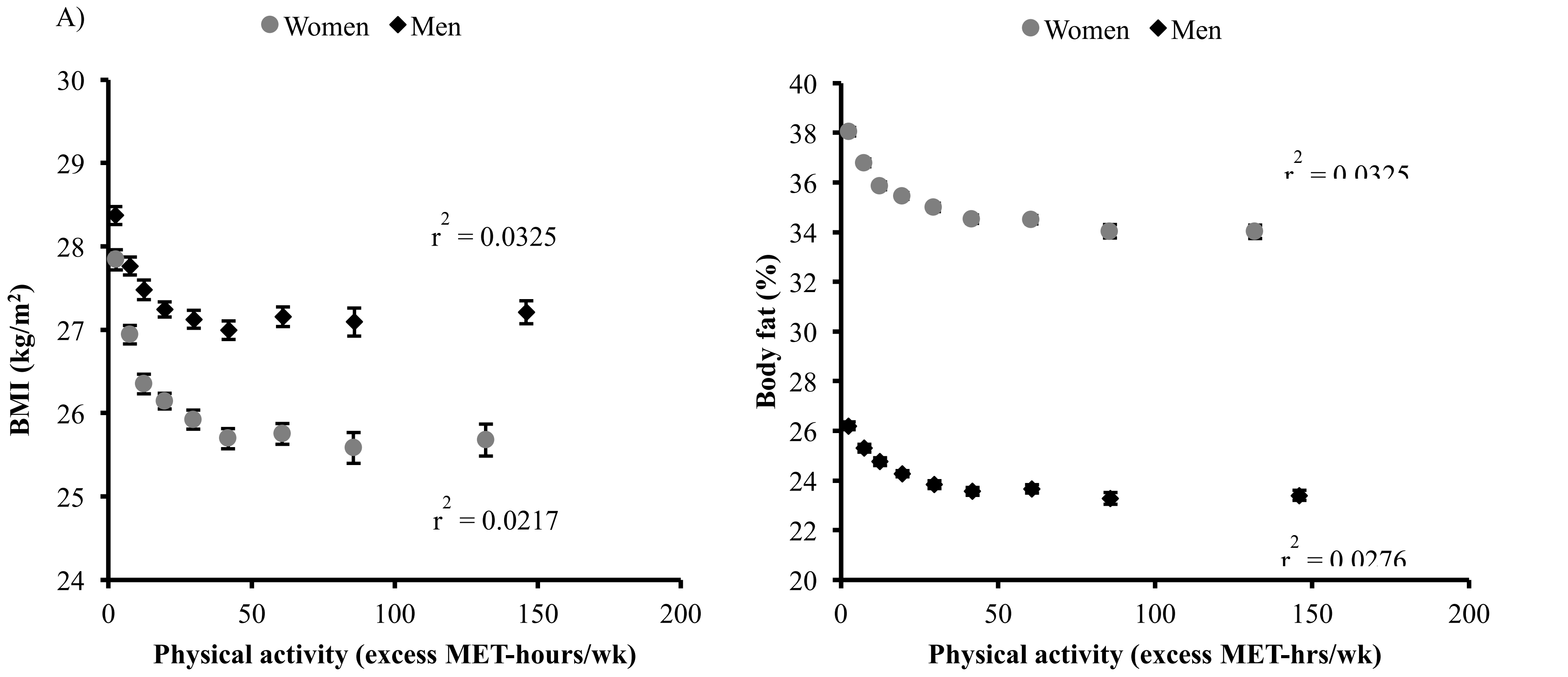


**Figure S5** Mean BMI and body fat percentage by physical activity in participants aged 50-59 years in UK Biobank

Panel A: Mean BMI by physical activity (excess MET-hours/wk); Panel B: Mean body fat percentage by physical activity (excess MET-hrs/wk)

Values are mean BMI and body fat percentage in the following categories of physcial activity: <5, 5-9.9, 10-14.9, 15-24.9, 25-34.9, 35-49.9, 50-74.9, 75-99.9, ≥ 100 excess MET-hrs per week, and are plotted at the value of the mean excess MET-hours/wk in each category

Errors bars are 95% CI

Estimates shown for cells with 200 or more participants

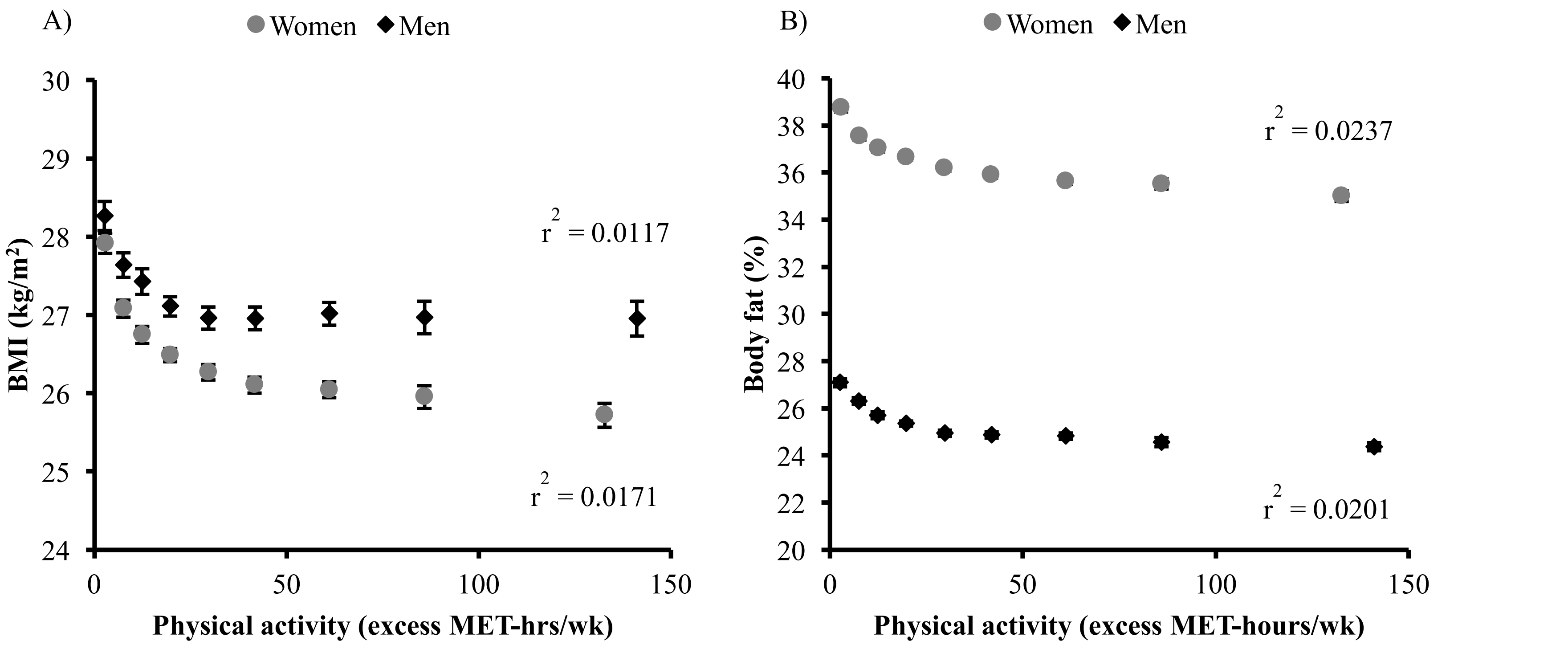


**Figure S6** Mean BMI and body fat percentage by physical activity in participants aged 60 years or older in UK Biobank

Panel A: Mean BMI by physical activity (excess MET-hours/wk); Panel B: Mean body fat percentage by physical activity (excess MET-hrs/wk)

Values are mean BMI and body fat percentage in the following categories of physical activity: <5, 5-9.9, 10-14.9, 15-24.9, 25-34.9, 35-49.9, 50-74.9, 75-99.9, ≥ 100 excess MET-hrs per week, and are plotted at the value of the mean excess MET-hours/wk in each category

Errors bars are 95% CI

Estimates shown for cells with 200 or more participants
